# *GSE*’s 50th anniversary: where do we go from now?

**DOI:** 10.1186/s12711-019-0504-4

**Published:** 2019-11-19

**Authors:** Genetic Selection Evolution’s Editorial Board

**Affiliations:** Paris, France

This year, *Genetics Selection Evolution* (*GSE*) celebrates its 50th anniversary. Indeed, it was in 1969 that the French National Institute for Agricultural Research (INRA) created the journal “*Annales de Génétique et de Sélection Animale*”, which was renamed “*Génétique Sélection Evolution*” in 1983, and translated into the current English title in 1989. Initially created to disseminate INRA’s academic and applied research in animal genetics, from the 1980s the journal aimed at extending its international influence and recognition under the impetus of its editors Jean-Louis Foulley and Francis Minvielle (see complete list of *GSE*’s editors-in-chief at the end of the article). During the last 20 years, *GSE* has established itself as one of the premier international scientific journals in its field, with currently at least half of the editorial board and two editors-in-chief of non-French origin, and more than 80% of the articles from non-INRA authors. *GSE* has been instrumental in developing quantitative genetics applied to farm animals, but also population genetics, including model species such as Drosophila under the leadership of Jean David (editor for *GSE*), whose research is dedicated to understanding the mechanisms of evolution using this model. Milestone papers include theoretical work on the properties of BLUP related to selection, methods for estimating variance components by REML, the use of Bayesian methods for parameter estimation, the development of reaction norm models, computation algorithms for genetic evaluation and parameter estimation, of non-linear models for categorical traits or survival analysis, of QTL mapping methods, and of genomic prediction models, as well as methods for prediction of rates of genetic improvement and inbreeding. The journal also published many applied research articles, among which those on estimates of genetic parameters, the first large-scale genetic diversity analyses of animal populations based on microsatellite data, QTL detection analyses, and the characterization of several major genes in different species. From the 2000s, the field of animal genetics was revolutionized by the emergence of SNPs and *GSE* published an early visionary article by Vignal et al. entitled “*A review on SNPs and other types of molecular markers and their use in animal genetics*” (*Genet Sel Evol*. 2002;34:275–305), followed by numerous articles on genomic prediction and selection.

With the changes in scientific publishing in the last 15 years, *GSE* has strived to extend its readership, first by releasing a free online version of its archives in 2006, and second by switching to Open Access with BioMed Central as publisher as early as 2009. These changes have taken *GSE* to a new level with a clear increase in both the number of articles submitted and published and in its impact factor; since 2012, it ranks among the top three journals in its category and has become the premier source of scientific literature on genomic prediction and selection.

Currently, the scientific disciplines that *GSE* targets are evolving fast. Major advances in technologies of genomic analysis on the one hand, and phenotyping methods on the other hand, are renewing current research in quantitative and population genetics. As in the past, *GSE* also benefits from developments in statistics and computing technologies. In the coming years, *GSE* aims to continue to consolidate its position as a leader in the field of animal genetics by supporting pioneering contributions, including the following areas:

- The sequencing of entire genomes will be systematic and on a very large scale. Thus, we will have access to very large designs and datasets to analyze the genetic architecture of traits and build more universal prediction models than the current genomic prediction models that are primarily valid only within a population.
- To better target genetic variants of interest and predict their interactions, statistical models for analysis of quantitative traits will be extended to integrate more biological information, which often consists of heterogeneous data, in particular, biological, functional, or more generally “Omics” data. To use such information and decipher its complexity, present statistical models used for genetic analysis may be challenged by prediction methods based on machine and deep learning.
- The contribution of two new major sources of information will be integrated into models for the analysis of trait architecture: on the one hand, contributions from studies on non-additive genetic effects and on intra- and trans-generational epigenetics, and on the other hand, contributions from studies of the microbiome (metagenomics). Future studies will show whether these two sources can improve the prediction of quantitative phenotypes and measure similarity between individuals.
- The availability of large amounts of phenotypes (thousands or more per animal), e.g., spectra (in milk, blood, etc.), automatic feed consumption recording, kynetics (with accelerometers), milk flow in milking robots, imaging data for animal tracking and behavior, GPS data and pregnancy test outcomes in cattle raised in extensive conditions, abattoir measurements of carcass quality, etc., will foster the need to develop new analytical methods for precision farming.
- To date, animal genetics has relied mainly on the characterization and use of the natural genetic variation that exists in populations. The potential of genome editing is considerable for understanding how the genome works, as well as understanding the genetic control of traits. If accepted by consumers and society, it can also have a major impact on breeding programs by being able to create new variation.
- In the last decade, contributions to *GSE* that focus on aquaculture have increased. In the coming years, the list of agriculturally relevant animal species covered by *GSE* will expand to include insects. Articles on plant genetics are also welcome when they introduce methods of interest for *GSE*’s readership. These will bring new challenges because of their population structures and reproductive characteristics.
- To face up to the new challenges of livestock farming, in particular agro-ecological challenges, renewed selection objectives are targeted. They have already evolved considerably over time by integrating an increasing number of traits, but we need to go even further to produce animals that are more resilient, more resistant to disease, and better adapted to expected global changes and to more sustainable production systems. Rather than searching for the best genotype to be disseminated in all the environments, we must identify robust genotypes that are adapted to each environment by better understanding, predicting, and capitalizing on genotype × environment interactions.
- While it was initially hoped that genomic selection would provide a means of better managing diversity, it appears that this is not always the case and that biodiversity may be faced with faster erosion. Thus, new approaches are needed to address the long-standing issue of the trade-off between selection and management of diversity. In addition, in contrast to present methods of genomic prediction, which are especially adapted to large populations, future methods must better take advantage of local genetic resources to characterize and use, and thereby preserve them.

The journal’s editorial team would like to sincerely thank all authors of publications in *GSE* for their valuable contributions to the scientific field in Genetics, Selection and Evolution, and is looking forward to publishing the next-generation of scientific discoveries in these areas.

*GSE’s editorial board*

## GSE’s editors in chief since 1969

- Jean-Jacques Lauvergne (1969–1982)
- Jean-Louis Foulley (1983–1985)
- Jacqueline Vu Tien Khang (1986–1988)
- Claude Chevalet (1989–1990)
- Louis Ollivier (1991–1996)
- Jean-Jacques Colleau (1997–1999)
- Francis Minvielle (2000–2005)
- Hélène Hayes (from 2006)
- Philippe Baret (2006–2011)
- Didier Boichard (from 2010)
- Jack CM Dekkers (from 2012)
- Julius HJ van der Werf (from 2015)

## Editorial Board

### Editors-in-Chief

- Dr. Didier Boichard, *French National Institute for Agricultural Research (INRA), France*
- Prof. Jack Dekkers, *Iowa State University, United States of America*
- Dr. Helene Hayes, *French National Institute for Agricultural Research (INRA), France*
- Prof. Julius van der Werf, *University of New England, Australia*

### Associate Editors

- Prof. Paolo Ajmone Marsan, *Università Cattolica del S. Cuore, Italy*
- Prof. Jörn Bennewitz, *University Hohenheim, Germany*
- Prof. Henk Bovenhuis, *Wageningen University, Netherlands*
- Prof. Armando Caballero, *Universidad de Vigo, Spain*
- Prof. Mario Calus, *Wageningen UR Livestock Research, Netherlands*
- Prof. Rodolfo Cantet, *University of Buenos Aires*-*CONICET, Argentina*
- Dr. Hans D Daetwyler, *La Trobe University, Australia*
- Prof. Andrea Doeschl-Wilson, *The Roslin Institute, University of Edinburgh, UK*
- Dr. Vincent Ducrocq, *French National Institute for Agricultural Research (INRA), France*
- Dr. Frederic Farnir, *University of Liege, Belgium*
- Prof. Martien Groenen, *Wageningen University, Netherlands*
- Prof. Bernt Guldbrandtsen, *Aarhus* *University, Denmark*
- Prof. Lusheng Huang, *Jiangxi Agricultural University, China*
- Prof. Dr. Christa Kuehn, *Farm Animal Biology Dummerstorf, Germany*
- Dr. Andres Legarra, *French National Institute for Agricultural Research (INRA), France*
- Dr. Ricardo Pong-Wong, *The Roslin Institute, UK*
- Dr. Bertrand Servin, *French National Institute for Agricultural Research (INRA), France*
- Dr. Tad Sonstegard, *Recombinetics, St. Paul, Minnesota, USA*
- Prof. Ismo Strandén, *Natural Resources Institute Finland, Finland*
- Dr. Toni Reverter, *CSIRO Agriculture & Food, Australia*
- Dr. Daniel Vaiman, *INSERM, French National Institute of Health and Medical Research, France*
- Dr. Roger Vallejo, *ARS National Center for Cool and Cold Water Aquaculture, USA*
- Dr. Alain Vignal, *French National Institute for Agricultural Research (INRA), France*
- Dr. Martine Yerle-Bouissou, *French National Institute for Agricultural Research (INRA), France*

